# Magnetisation Transfer 3D-Radial Zero Echo Time MR Imaging at 7T

**DOI:** 10.3390/jcm14217722

**Published:** 2025-10-30

**Authors:** Mark Symms, Paulina Kozioł, Catarina Rua, Douglas Kelley, Natalia Pietroń, Katarzyna Wiśniewska, Anna Niedziałek, Anna Jamroz-Wiśniewska, Andrzej Stepniewski, Radosław Pietura

**Affiliations:** 1 Independent Researcher, Gerrards Cross, Buckinghamshire SL9 0RJ, UK; markrogersymms@outlook.com; 2 Department of Radiography, Medical University of Lublin, 20-093 Lublin, Poland; paulina.koziol@op.pl (P.K.); anna.niedzialek@umlub.edu.pl (A.N.); 3 Antaros Medical, 43183 Mölndal, Sweden; catarina.h.s.rua@gmail.com; 4 Department of Radiology, Stanford School of Medicine, Stanford, CA 94305, USA; kelleyda@stanford.edu; 5 Department of Diagnostic Imaging, University Hospital No1 in Lublin, 20-400 Lublin, Poland; natalia.nieradko.96@gmail.com (N.P.); katarzyna.agnieszka.wisniewska@gmail.com (K.W.); 6 Department of Neurology, Medical University of Lublin, 20-093 Lublin, Poland; anna.jamroz-wisniewska@umlub.edu.pl; 7 ECOTECH-COMPLEX, Maria Curie-Skłodowska University in Lublin, 20-031 Lublin, Poland; astep@ipan.lublin.pl

**Keywords:** magnetisation transfer, zero echo time MRI, multiple sclerosis

## Abstract

**Background/Objectives**: Magnetisation Transfer (MT) MRI is used for neuro-degenerative disorders, including Multiple Sclerosis (MS), providing an indirect measure of large biomolecular MR signal sources which cannot be observed directly because their typical T2 is usually much shorter than the echo time (TE) of conventional MR sequences. We investigated a 3D-radial Zero Time of Echo (ZTE) MT-weighted sequence with potentially enhanced sensitivity to short-T2 MR signals indirectly (via MT weighting) and directly (due to the short TE). **Methods**: The sequence runs on a human 7T MR scanner, producing whole-brain MT-weighted images with isotropic 0.8 mm resolution in 6.5 minutes. One RF pulse is used to suppress the fat signal and generate MT weighting, reducing RF power deposition to moderate levels. The small excitation pulses and the “quasi-adiabatic” MT pulse mitigate the negative effects of inhomogeneous transmit RF fields observed at 7T in the human head, facilitating the generation of uniform Magnetisation Transfer Ratio (MTR) maps. **Results**: Results from a biologic phantom, a healthy volunteer, and an MS patient illustrate important imaging features of the “SilentMT” sequence. When the MS patient images were compared with Fluid Attenuated Inversion Recovery (FLAIR) images taken on the same patient at 1.5T and 7T, SilentMT was able to detect all the MS lesions observed on the “reference truth” 1.5T FLAIR; 7T FLAIR, however, failed to detect some lesions in the temporal lobe and brain stem. SilentMT detected a lesion which was not immediately apparent on either FLAIR image. Increased MTR was observed in some regions of the brain of the MS patient, notably the left temporal lobe. **Conclusions**: This initial investigation of an MT-weighted ZTE sequence shows evidence that it may be more sensitive to pathology in a patient with MS.

## 1. Introduction

Magnetic Resonance Imaging (MRI) has been used for the study of brain diseases such as Multiple Sclerosis (MS) for 40 years [1]. The 2010 McDonald criteria for the diagnosis of MS are frequently used in both clinical and research spheres, and are regularly updated [2]. A full list of recommended sequences for 1.5 and/or 3T MR scanning of MS patients [3,4] includes T2-weighted Fast Spin Echo (FSE), sagittal T2-FLAIR, and an axial T1-weighted image following injection of Gadolinium contrast agent.

With the recent introduction of 7T MR scanners with regulatory approval as medical devices, a consensus is now beginning to form on recommendations for the best use of 7T MR for MS [5]. To the above 3T list, they add MP2RAGE (for atrophy, segmentation, and lesions), 3D FLAIR, and whole-brain multi-echo Gradient-Echo with T2* contrast.

However, there are known limitations in MR signal detectability which may have important diagnostic consequences. Reception of the MR signal is constrained by the delay between the transmission of the excitation pulse and the time it takes to switch the scanner into receive mode. This can be limited by how long the receive coil takes to be readied for reception or how quickly the MR scanner receive electronics can recover following the transmit pulse, but the shortest achievable TE is often determined by the performance of the MR scanner gradients and is typically in the region of 5 ms.

The MR signal is usually understood to decay exponentially according to the transverse relaxation time T2 or T2*. Mathematically, 98% of the signal following an exponential decay has gone after five times the relaxation time (or more generally, “half-life”). This means MR signals with a T2 less than 1ms decay too rapidly to be detected by the scanner using a sequence with a TE of 5ms.

Citing measurements made by Wilhelm et al. [6], Weiger et al. [7] show that many of the components of neuronal myelin have T2 values which cannot be detected by most MR scanners operating today. It is therefore quite possible that MR sequences currently recommended for MS running on commercial clinical MR scanners may be unable to detect important changes occurring in the myelin known to be associated with many neurodegenerative disease processes. This is reminiscent of the “dark matter” problem in astronomy, where scientists making observations of objects they could see in the night sky realised those objects were experiencing gravitational interactions with matter which they could not directly observe with their instruments.

MT (Magnetisation Transfer) contrast has been used to indirectly probe short-T2 MR signals by those who are interested in large biomolecular systems [8,9] and those studying neurodegenerative disorders with MR. Radiofrequency (RF) radiation is applied 1 kHz or more away from the MR resonance, saturating the less mobile large biomolecule MR signal. As this “solid pool”—which is invisible to direct detection by the scanner due to its short T2—is in exchange with the “mobile pool” of water protons, magnetisation transfer occurs, attenuating the detected mobile proton signal.

By taking the ratio between an image affected by MT and a similar image without MT weighting, an MTR map can be generated. MTR maps have been used in the study of MS for decades [10]. Reduced MTR is known to be associated with demyelination and increasing (or normalising) MTR a sign of remyelination [11]. MTR is very reproducible when used on a single MR scanner; on the other hand, multi-site and multi-vendor studies pose considerable technical challenges [12]. MTR maps have a very flat, even contrast; their value as a biomarker comes from the fact that relatively small changes are usually highly significant compared to the uniform background.

Pathology studies of brains with MS have shown that disease progression varies across the brain [13], showing more changes at and near the cortex and close to the ventricles. Some MR imaging research groups [14,15,16] have developed complex post-processing techniques to analyse MTR maps of MS patients and they give evidence of an “MTR gradient” across the brains of patients with MS which seems to correlate with disease activity and type of MS.

ZTE sequences [17,18] and the closely related Ultra-short TE (UTE) class of sequences [19,20,21] have many similar properties; both are designed to minimise the echo time (or for FID sequences, the time to first data acquisition following excitation) to the extent that short-T2 NMR signals can be directly observed. In previous years, UTE sequences usually had a longer minimum TE than ZTE sequences, but improvements in gradient strength and stability, as well as faster RF electronics and coils, mean that UTE performance can now match ZTE sequences; Ma et al. [22] quote a TE of 32 μs, which is very similar to that achieved with ZTE sequences such as Silent [23].

Ljungberg et al. [18] make the distinction that ZTE sequences apply the excitation pulse while the imaging gradients are already on, whereas the typical UTE schemes [20] place the first gradient ramp following RF excitation.

Several groups have investigated the use of UTE imaging to detect short-T2 MR signals [22,24,25]. UTE has also been used to generate MR images of other short-T2 spins, as found in lung and cartilage imaging [26]. UTE usually has a minimum TE determined by the slice-selection gradient in the sequence, though it is possible to use hard (non-selective) RF excitation pulses instead [27].

Baadsvik et al. [28] have developed a scanner and sequences capable of running ZTE sequences with very short TEs, which are intended for the direct detection of short-T2 MR signal such as that observed in myelin.

The “Silent” MR sequence has been in development by scientists in GE HealthCare and their collaborating customers for the past 15 years [18,29,30]. The sequence is a three-dimensional version of a ZTE MR sequence 2D-RUFIS [17]. It uses short intense rectangular hard RF excitation pulses in combination with RF coils (and other associated RF components) capable of rapid (<20 μs) switching between transmit and receive modes to permit the acquisition of MR signal very quickly (typically 40 μs) after RF excitation [18].

Various groups have been extending the application of GE’s Silent sequence at 7T. Early images obtained with T1-weighted Silent [31] demonstrated that the sequence could detect objects—such as the pads supporting the patient’s head—which could not be seen on conventional MR images acquired at the same time with the same scanner.

The same group [32] described a ZTE sequence which showed strong MT (Magnetisation Transfer) contrast but was limited by high RF power deposition and had poor spatial resolution. The sequence used an “ASPIR” (Adiabatic Spectral Inversion Recovery) pulse which performs the dual functions of fat suppression and imbuing Magnetisation Transfer contrast. The fat–water frequency separation for a traditional “CHESS” fat suppression pulse is about 1100 Hz at 7T, which is also a good offset for Magnetisation Transfer; smaller offsets have much higher direct saturation [33]. More recently, others have exploited this effect to perform MT-weighted Silent at 7T in the eyeball [34].

The MT and UTE/ZTE approaches both allow the observation of short-T2 MR signal. The observation of short-T2 signal by MT is indirect, while UTE/ZTE sequences can directly acquire signals with short T2. Tyler et al. [18] discussed cases where short echo time imaging sequences such as UTE could be combined with Magnetisation Transfer contrast. They pointed out that weighting a conventional Gradient Echo sequence with MT explores the exchanges between a long-T2 pool (T2 = 50–100 ms) and a short-T2 pool (T2 = 10–250 μs). They suggested that MT-weighted UTE could investigate the exchanges between a short-T2 pool (T2 ~ 500 μs) and a very short-T2 pool (T2 < 10 μs), thus providing new information about this very short-T2 pool which may not be obtained with current techniques.

As some short-T2 components of myelin fall within the range of 10 μs [7], it is therefore possible that MT-weighted Silent, which has a similarly short TE, and MTR maps derived from two Silent images may include extra information about the state of myelinated cells not currently observable with other techniques.

Although 7T MR has a higher inherent SNR than lower field MR, 7T images are often adversely affected by several problems. The stronger main field generates susceptibility effects, leading to image distortions and signal drop-outs. The receiver coil array has an inhomogeneous reception field, causing signal from regions of the brain closer to the coil to be more intense. There are also strong variations in the transmit field (B1+), including a gradual decrease in B1+ from the top of the head to the neck and also a very significant variation across any slice taken from this area, because the head at 300 MHz acts as a resonant cavity for the RF [35]; variations of 300% are typically observed. RF power deposition is much higher at 7T [36], meaning sequence parameters and timings are often limited by RF power constraints. RF power is transmitted to the head using a small volume birdcage coil rather than a whole-body coil, leading to a subject-dependent variation in transmit B1 in the temporal lobes.

Most MR images at 7T are affected by these problems. All images are affected by receive coil array inhomogeneity. This effect can be mitigated image-based bias correction routines such as N4ITK [37]. EPI and Gradient-Echo images are affected by susceptibility effects, leading to geometric distortions. Spin Echo sequences are affected by B1+ because a 300% variation can turn a nominal 60-degree excitation pulse into a 180-degree excitation or vice versa, causing large changes in image contrast and lesion conspicuity.

Spin echo sequences are also limited by RF power deposition, as are Magnetisation Transfer-weighted sequences, which normally have high RF power deposition.

As MT weighting is proportional to B1+, MT images and maps also exhibit a “doming” effect across a central slice of the brain. MT sequences offered commercially by the vendors are somewhat limited in performance, which has in some cases prompted alternative efforts to acquire MT-weighted images [38]. This non-uniformity of MT weighting drastically reduces the quantitative value of MT and limits its applicability to serial imaging at 7T.

Several approaches have been made to address the issue of B1+ inhomogeneity. Khlebnikov et al. [39] used two different methods to correct their 7T quantitative CEST measures but required an additional measure of B1 as well as building some assumptions into their post-processing models. Schuenke et al. [40] and Herz et al. [41] used adiabatic spin-lock pulses [42,43] in a modified CEST acquisition at 7T and 9.4T to reduce effects of RF field inhomogeneity. Miller et al. [44] designed a “quasi-adiabatic” RF pulse to perform a saturation transfer experiment in an animal 9.4T scanner with similar B1 inhomogeneities, demonstrating greatly reduced sensitivity to B1 inhomogeneity.

Silent is well suited to addressing the challenges of inhomogeneous B1 and B0 fields at 7T. The hard excitation pulses have very low flip angles, so contrast changes due to B1+ changes are reduced. The lack of a slice-selection module reduces drop-out artefacts. Silent is affected by susceptibility gradients caused by the 7T field, but much less so than EPI or high-resolution Gradient-Echo imaging [18]. Silent also has relatively modest RF power, making it safe and reliable in routine clinical use [18,31].

We have modified the MT-weighted Silent sequence previously reported [32] so it has a longer “spoke-train” (3D-radial equivalent of echo-train), lower RF power deposition, and improved resolution [45].

The sequence uses an ASPIR pulse (provided as part of the GE product pulse library) to perform both fat suppression and provide MT weighting [32]. The ASPIR pulse is a frequency-swept spin-locked pulse [42,43], so does not have a pure, well-defined frequency offset, but most of its power is applied at its nominal offset frequency [32].

The ASPIR pulse’s adiabatic quality has the important benefit of providing MT weighting with improved robustness against B1 inhomogeneity, just as Miller et al.’s “quasi-adiabatic” pulse does for saturation transfer [44].

A commonly-used phantom for short T2 studies is egg, cooked to varying degrees [46]. Egg can also be used as a Magnetisation Transfer phantom [47].

This work performed scans on humans and an egg phantom to investigate three properties of SilentMT: uniformity of MT weighting, short-T2 detection, and radiologic performance.

(1) Uniformity of MT weighting

Our first aim was to demonstrate the robustness of the ASPIR pulse against B1+ inhomogeneity in a human scan by comparing the uniformity of an MTR map generated from the ASPIR pulse with an MTR map generated from a sequence that used a rectangular “block” MT-weighting pulse.

(2) Short-T2 detection

Secondly, we investigated whether MT-weighted 3D-radial imaging (“SilentMT”) can detect changes in an MS patient and a biologic egg phantom which are not detected with conventional MR sequences.

(3) Radiologic performance

Finally, we evaluated Silent images for MS lesion detection by comparing SilentMT with images taken in the same session with a prototype FLAIR sequence optimised for 7T [48] and with FLAIR images taken at 1.5T within two weeks of the 7T scan as a “reference truth.” One of the first applications of FLAIR was the detection of MS lesions [49], and FLAIR is widely recommended for diagnosis of MS at 1.5T and 3T [4], as well as 7T [5].

We also reviewed the images and maps generated from the SilentMT to see if its enhanced signal acquisition scheme (direct + indirect) would reveal any new possibly relevant imaging features.

## 2. Materials and Methods

### 2.1. Human Scanning

A healthy volunteer and a volunteer with Multiple Sclerosis were scanned. All participants signed informed consent. This research was approved by the local ethics committee of the Medical University of Lublin (KE-0254/118/2021).

At 7T, the healthy volunteer had SilentMT and a similar Silent scan without MT weighting.

The MT-weighted 2D Gradient-Echo sequence provided by the vendor was also used to create an image with MT weighting and a similar image without MT weighting. A B1 map was acquired at the same location as the 2D Gradient-Echo MT scan.

These scans were used for the “Uniformity of MT-weighting” tests and as a control comparison for the MS patient scans.

At 7T, the volunteer with MS had SilentMT and the same Silent scan without MT weighting and a FLAIR scan.

At 1.5T, within two weeks of the 7T scan, the volunteer with MS had a 1.5T FLAIR scan.

These scans were used for the “Radiologic performance” assessments.

### 2.2. Biologic Egg Phantom Scanning

An egg was boiled for 10 min, then left at room temperature for 24 h.

We scanned the egg with a SilentMT scan and an unweighted Silent scan, with parameters identical to the human scans.

We also scanned the egg with a 3D Gradient-Echo sequence at the same location as the Silent scan using similar parameters and an ASPIR fat suppression pulse. Following the methodology of Rua et al. [38], we used signal from the egg white to calibrate the MT weighting of this Gradient-Echo sequence so it was close to that obtained by the SilentMT scan.

A small 10 cm diameter sphere of water was scanned at the same time as the egg to provide a within-image reference with neutral contrast and to confirm correct registration between the two sequences.

These scans were used for the “Short-T2 detection” comparison.

### 2.3. Scanners and Sequence Details

All 7T scans were performed on a General Electric 7T MR950 (7T23.v03b software) MR scanner (GE HealthCare, Waukesha, WI, USA) using a Nova Medical 2-channel head birdcage coil for RF transmission and a Nova Medical 32-channel array coil for reception (Nova Medical, Wilmington, MA, USA).

The 1.5T scans were performed on a General Electric 1.5T Signa Artist MR scanner (MR29 software) (GE Healthcare, Waukesha, WI, USA) using the body birdcage for transmission and the vendor’s head/neck neurovascular (“Head-Neck Unit”) coil for reception.

**Figure 1:**
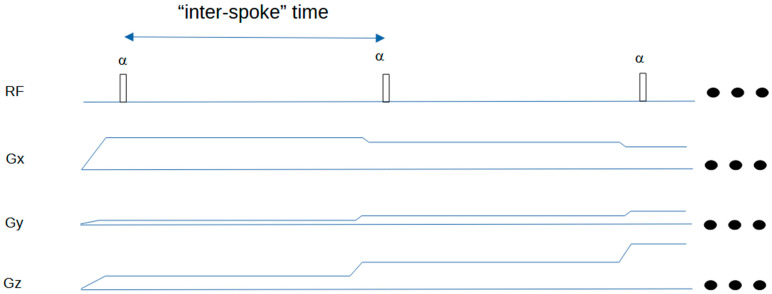
Timing diagram for Silent sequence. The first two spokes of a typical 3D-radial sequence are shown. The FID signal is acquired following each RF excitation pulse. The three dots on the right indicate that the sequence then repeats. The “inter-spoke” time can be taken as a repetition time for MR signal evolution calculations in a spoke-train.

**Figure 2:**
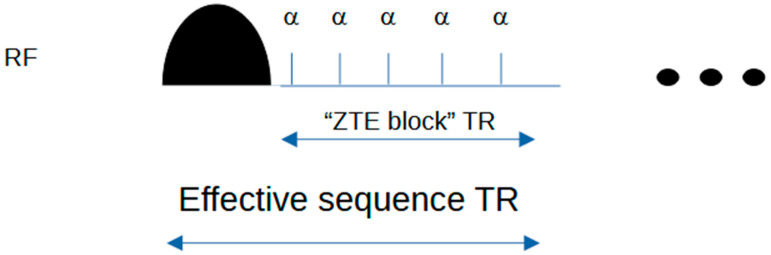
Silent timing diagram showing intermittent fat suppression scheme. The ASPIR fat suppression pulse is followed by multiple RF excitation pulses and associated gradient spokes (not shown). The prototype sequence reports the “ZTE block” time as TR; the effective (true) TR is approximately 25 ms longer when the time for the Chemical Saturation pulse is included. Both are defined in this figure. Note that the pulse widths are not to scale: ASPIR pulse width 20 ms, excitation pulse width 20 μs. Usually, about 100 spokes are acquired following each fat suppression pulse. The three dots on the right indicate that the sequence then repeats.

A schematic of the Silent sequence is shown in Figure 1 for a simple 2-spoke example. Figure 2 shows how the ASPIR fat suppression/MT-weighting pulse is applied in an intermittent fashion, before the acquisition of a train of spokes to create the Silent image.

#### Sequence Parameters

The SilentMT parameters were: TE 40 μs, inter-spoke time 2.3 ms, “ZTE block TR” 257 ms, effective TR 282 ms, matrix 224 × 224 × 224, field of view 17.6 cm, voxel size 0.8 mm isotropic, number of excitations 3, spokes/segment 224, spokes per chemical saturation pulse 112, scan time 6 min 30 s, ASPIR spin-locked Hyperbolic Secant fat suppression pulse: pulse width 20 ms, flip angle 180 degrees.

Immediately after the SilentMT scan, a second Silent scan was taken at the same location using identical transmit and receiver gain settings. The fat suppression pulse was changed from ASPIR to the standard “CHESS” pulse. The CHESS pulse (pulse width 3.2 ms, flip angle 90) suppresses the fat signal, but is a much shorter, less intense pulse which should not induce significant MT weighting. All other parameters were the same as the SilentMT scan. We call this the unweighted Silent scan. The SilentMT scan and the unweighted Silent scan were used to calculate the MTR map (details below).

For the volunteer with MS, we additionally acquired 7T FLAIR with the following parameters: 3D-CUBE FLAIR, TE 103 ms, TR 10 s, TI 2383 ms, matrix 224 × 200, field of view 22.4 × 20 cm, isotropic voxel size 1 mm, number of excitations 0.75, echo-train length 240, scan time 8 min 34 s.

We also acquired 1.5T FLAIR with the following parameters: 3D-CUBE FLAIR, TE 98 ms, TR 6.8 s, TI 1752 ms, matrix 256 × 256 (reconstructed to 512 × 512), field of view 25.6 cm, voxel size 1 mm × 1 mm × 0.7 mm, slice thickness 1.4 mm (interpolated to 0.7 mm), echo-train length 240, number of excitations 1, scan time 8 min.

All the images described above were acquired in the sagittal orientation.

For the healthy volunteer, we acquired SilentMT and unweighted Silent scans (see above for parameters).

We acquired an axial MT-weighted 2D Gradient-Echo sequence with a block (rectangular) MT pulse and a 2D Gradient-Echo sequence with identical parameters and instrument settings, except the amplitude of the MT pulse was set to zero, giving an unweighted 2D Gradient-Echo image. These two images were used to calculate an MTR map (details below) used in the “Uniformity of MT weighting” assessment.

The Gradient-Echo MT-weighted sequence parameters were: 2D Fast Gradient Echo, TE 3 ms, TR 200 ms, flip angle 2 degrees, single slice thickness 5 mm, in-plane matrix 128 × 128, field of view 20 cm, scan time 30 s, MT pulse width 8 ms, flip angle 500 degrees.

We acquired a Bloch-Siegert B1-mapping sequence [50] at the same axial location as the Gradient-Echo image. The Transmit Gain was set to the same value as in the Gradient-Echo sequence. The B1-mapping sequence parameters were: 2D Fast Gradient Echo, TE 10 ms, TR 100 ms, flip angle 2 degrees, single slice thickness 5 mm, field of view 20 cm, in-plane matrix 64 × 64, scan time 50 s, Bloch-Siegert pulse width 8 ms, offset +/− 4 kHz, flip angle 270.

For the egg phantom, we acquired SilentMT and unweighted Silent scans similar to those taken in the human scans.

For the “Short-T2 detection” evaluation, we also acquired a 3D Gradient-Echo MT-weighted sequence (using an ASPIR fat suppression pulse) with coverage and resolution matching the SilentMT scans. Parameters for the 3D Gradient-Echo scans were: 3D Spoiled Gradient Echo with ASPIR fat suppression, TE 3 ms, TR 7.7 ms, flip angle 2, matrix 224 × 224 × 224, field of view 17.6 cm, slice thickness 0.8 mm, scan time 3.5 min.

### 2.4. MTR Map Calculation

The Magnetisation Transfer Ratio calculation requires an MT-weighted image (“S_MT_”) and an unweighted image with similar scan parameters and instrument settings (“S_UW_”).

The Magnetisation Transfer Ratio (MTR) is then calculated on a voxel-by-voxel basis using the fslmaths routine from FSL [51]:

MTR = (1 − S_MT_/S_UW_) × 100 (expressed in percentage units)

### 2.5. MS Patient Image Processing and Review

For the MS patient, the images were registered to the SilentMT image using FLIRT [51] with 12 degrees of freedom (affine transformation) and a normalised mutual information cost function. The FLAIR 7T image (which was acquired in the same space in the same session as the Silent image by copying the graphic prescription) was registered directly to the Silent image. The FLAIR 1.5T image was then registered to the FLAIR 7T image which had previously been registered to the Silent image. As the unweighted Silent image had been acquired immediately after the SilentMT image using the same graphic prescription, visual side-by-side inspection validated that these two images were in close register. This approach placed all images in the same physical space with identical matrix sizes and voxel resolutions, allowing straightforward comparison using the multiple display function available in ITK-SNAP [52]. Visual inspection of several anatomical landmarks suggested the images were within a voxel (0.8 mm) of perfect alignment.

An experienced neuroimaging researcher reviewed the registered 1.5T FLAIR, the registered 7T FLAIR, and the SilentMT images from the MS patient in a side-by-side review using ITK-SNAP [52]. The entire volume was scrolled through axially and inspected slice by slice. On each slice, a visual assessment was made as to whether each apparent MS lesion visible on the 1.5T FLAIR could be seen on the 7T FLAIR and on the SilentMT image. The locations of possible differences in lesion detection between the sequences were noted. Two radiologists then reviewed and confirmed these findings at these locations.

The Silent MTR map from the MS patient was visually reviewed, and compared to the Silent MTR map from the healthy volunteer.

### 2.6. Biologic Egg Phantom Analysis

Alignment between the two images was confirmed by comparison of the reference sphere’s position in the two images. As the sequence parameters of the Silent and Gradient-Echo images are similar, and the MT weightings are also similar in amplitude, the major significant difference between the two images is the echo time: ~40 µs for Silent and 4 ms for the Gradient-Echo image. The images were visually reviewed.

### 2.7. Uniformity of MT-Weighting Measurements

An MTR map was calculated from the two Silent images (which use the ASPIR pulse) in the healthy volunteer. A second MTR map was calculated from the 2D Gradient-Echo MT-weighted and unweighted images. The MTR for this map was encoded using the block pulse, which is not adiabatic.

The axial location of the Gradient-Echo MTR map was identified in the Silent MTR map using location information from the original source DICOM images. The relevant (reconstructed axial) Silent MTR map was compared with the MTR map from the Gradient-Echo sequence and with the B1 map in a side-by-side review using ITK-SNAP (University of Pennsylvania, PA, USA). Profiles along the left-right direction were measured to visually assess left-right uniformity in the two MTR maps.

## 3. Results

Uniformity of MT weighting: comparison of block MT-weighted pulse and ASPIR adiabatic pulse on MT-weighted image and MTR map uniformity.

**Figure 3:**
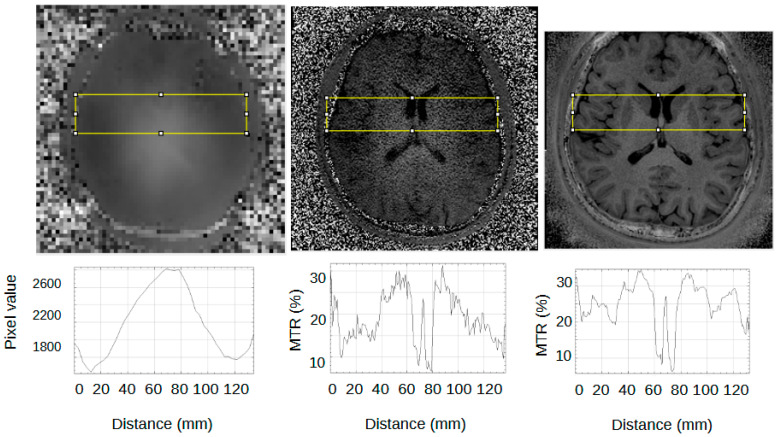
Effect of B1+ inhomogeneity on MTR. (**Top row**) Left B1+ map; Centre MTR using block MT pulse; Right MTR using ASPIR adiabatic pulse. (**Bottom row**) Profiles corresponding to areas shown in yellow boxes. The area from distance 100 mm to 120 mm on the *x*-axis is a region of strong left–right intensity gradient in the B1 map and in the MTR map derived from the block MT pulse, but not in the Silent MTR map derived from the ASPIR pulse.

Figure 3 shows the transmit B1 map, the MTR map from the product MTR sequence, and the MTR map from the Silent images using the ASPIR pulse. A gradient can be observed in the left-right direction from 100 mm to 120 mm on the distance axis in the B1+ map (left) and in the MTR map derived from the block MT pulse, but the Silent MTR map shows better visual left–right uniformity. Similar changes are seen in the profile of the MTR derived from the block pulse (centre) but not in the Silent MTR profile, which uses the adiabatic ASPIR pulse.

MT weighting correlates strongly with transmit B1. Note that this only applies to brain, not to CSF, which has no MT effect.

**Figure 4:**
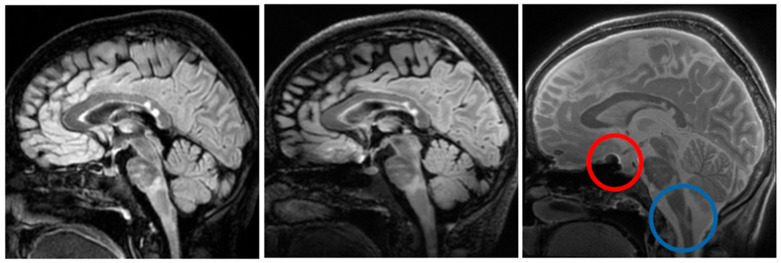
Brain-stem lesions. Brain-stem lesion (blue circle) observed on 1.5T FLAIR (**left**). FLAIR 7T contrast is strongly affected by transmit B1 inhomogeneity—MS lesion is very faint (**centre**). SilentMT contrast is robust against transmit B1 inhomogeneity—lesion observed (**right**). Red circle shows region affected by trajectory errors in SilentMT.

Visual Assessment of SilentMT image features:

Figure 4 shows an example sagittal slice from the MS patient through the brain-stem. The blue circle outlines a brainstem lesion which is visible on the SilentMT image and the 1.5T FLAIR image, but difficult to see on the 7T FLAIR. Just above the blue circle, another brain-stem lesion can be seen. The 1.5T and 7T FLAIR images show this lesion as brighter than other local features whereas on the SilentMT image, the lesion appears as bright as the neighbouring CSF. A qualitative visual assessment of the SilentMT image is that there is strong contrast between CSF, grey matter, and white matter, reminiscent of what is seen in T2-weighted images. Image contrast appeared relatively uniform across the cerebrum and cerebellum, and so less affected by the transmit B1 inhomogeneity typically observed in the head at 7T.

The Silent images showed some “trajectory error” geometric distortion in areas of strong B0 inhomogeneity such as can be observed at the base of the brain in Figure 4 (red circle).

An experienced neuroimaging researcher and two radiologists reviewed the registered 1.5T FLAIR, the registered 7T FLAIR, and the SilentMT images from the MS patient. A consensus was reached among the reviewers that the SilentMT image detected all the same MS lesions as the 1.5T FLAIR. We observed that while 7T FLAIR also detected the MS lesions in most slices, MS lesions were not always detected in the brain-stem and in the temporal lobes.

**Figure 5:**
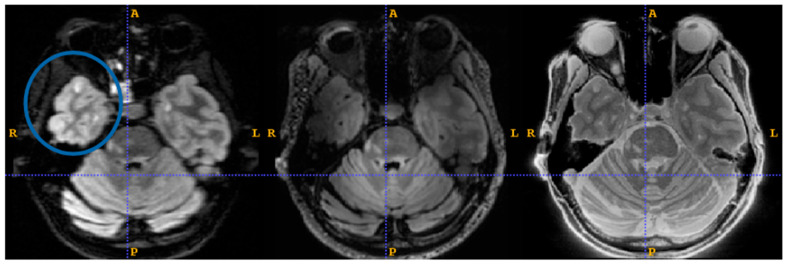
7T FLAIR contrast loss in left temporal lobe. MS lesions are visible (blue circle) on 1.5T FLAIR (**left**) and SilentMT (**right**). MS lesions are not visible on 7T FLAIR (**centre**).

Examples of lesions not being detected in the temporal lobe are given in Figure 5. In Figure 5, we count 3 lesions in the right temporal lobe (blue circle) which are visible on 1.5T FLAIR (left) and SilentMT (right), but are not apparent on the 7T FLAIR (centre). In the left temporal lobe, we can see 1 lesion on the 1.5T FLAIR and the SilentMT, but the same lesion is not visible on the 7T FLAIR.

**Figure 6:**
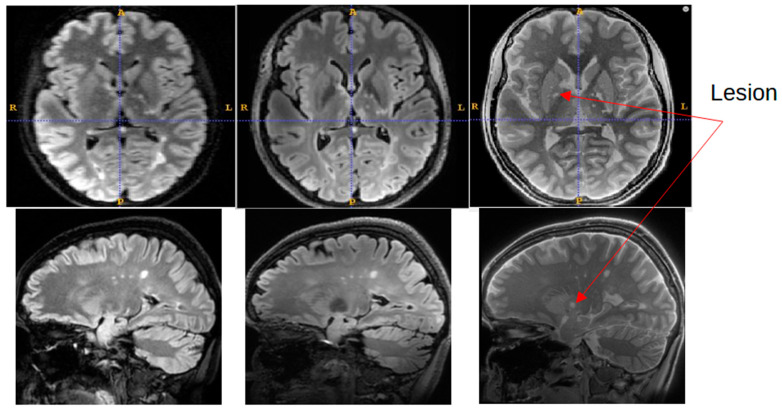
Lesion detected on SilentMT (red arrows) but not observed on axial 1.5T FLAIR or 7T FLAIR. Subsequent radiologic review showed the lesion with a faint and blurred appearance on 1.5T sagittal FLAIR.

Figure 6 shows a representative slice from the MS patient where an MS lesion detected with SilentMT was not immediately apparent on either FLAIR sequence. The lesion is located peri-ventricularly in a region likely to be clinically relevant for MS but was not initially detected on the FLAIR sequences. Subsequent radiologic review of this region reached the consensus that the lesion was not visible on the 7T FLAIR and was of such faint and blurred appearance on the 1.5T FLAIR that it could easily have been missed in a normal clinical review. The lesion was slightly more conspicuous on the 1.5T sagittal FLAIR image (the original plane of acquisition) than on the axial reformatted 1.5T FLAIR image.

**Figure 7:**
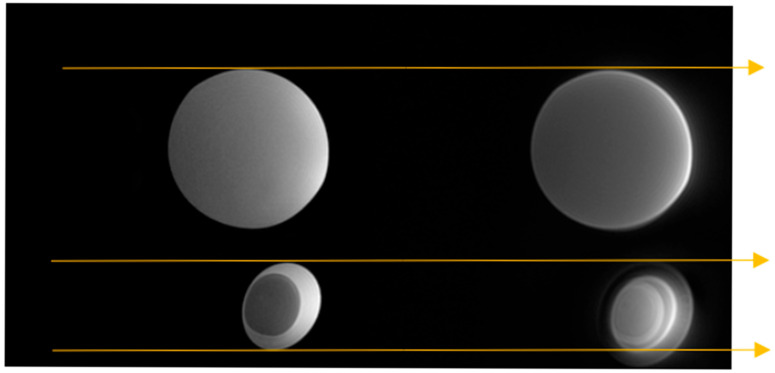
MT-weighted scans of a boiled egg. MT-weighted Gradient Echo (**left**) SilentMT (**right**). Horizontal gold guide-lines have been added to aid visual comparison.

Figure 7 shows the boiled egg phantom (lower) and a small water-filled sphere (upper) scanned with MT-weighted Gradient-Echo images (left) and MT-weighted Silent (right). The images were acquired in register in the same session, as the added guidelines (gold arrows) show. The Gradient-Echo image shows good contrast between the white and the yolk of the egg. The Silent image suggests a greater level of structure and detail in the same egg at the same slice location.

**Figure 8:**
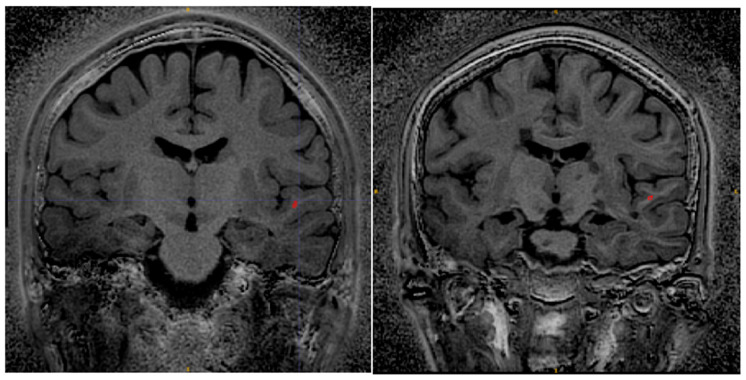
Silent MTR in healthy control (**left**) and MS patient (**right**). Silent MTR is increased in region of left temporal lobe and peri-ventricularly. ROI’s used to make MTR measures are shown in red.

Figure 8 shows Silent MTR maps from a healthy volunteer (left) and a patient with Multiple Sclerosis (right). The MTR map of the healthy volunteer shows some contrast between grey and white matter. The MTR of the white matter is fairly uniform across the brain of the healthy volunteer. The Silent MTR map of the MS patient shows visually apparent increases in MTR peri-ventricularly and in the left temporal lobe.

Regions of interest were drawn in equivalent locations of the superior temporal lobe in the MS patient and the control.

The MS patient ROI had mean MTR 37.5% and standard deviation 4% (percentage units). The control ROI had mean MTR 28.6% and standard deviation 1.5%.

## 4. Discussion

We have developed and optimised an MT-weighted ZTE sequence “SilentMT” for radiological imaging and semi-quantitative measurements of MTR at 7T. We obtained high-resolution MT-weighted Silent images at moderate levels of RF power deposition in a clinically useful scan time.

The transmit RF map shows the large variation typically observed at 7T in the human head. MT weighting depends strongly upon transmit B1, so the MTR map generated from a block MT pulse also displayed large variations in the slice. The MTR map generated using the adiabatic ASPIR pulse in SilentMT did not show large variations and was of uniform visual appearance. We conclude MTR derived from the ASPIR pulse is robust against transmit B1 inhomogeneities, as has previously been demonstrated by Miller et al. in a small animal scanner [44]. Some degradation of signal was observed at the inferior base of the temporal lobe, a familiar effect associated with the head-only Nova Medical transmit coil.

The robustness of SilentMT to B1+ changes means the images show uniform tissue contrast which extends throughout the brain and into the neck. A side-by-side visual review of images of an MS patient showed that SilentMT at 7T could detect all of the MS lesions that were picked up on the “reference truth” 1.5T FLAIR, though lesion brightness was lower in SilentMT than in FLAIR.

In the MS patient, 7T FLAIR seemed to have less diagnostic value in the brain-stem and the temporal lobes. This finding warrants further investigation. The RF pulses used in this prototype sequence require good B0-shimming for correct operation; if the shims were not well set during the 7T FLAIR scan, then we might expect sub-optimal results in the temporal lobes, which are known to have strong B0 inhomogeneity. The other explanation could be the effects of poor transmit RF field homogeneity, which are known to affect lesion and tissue contrast in long spin-echo trains used by sequences such as CUBE (used here) or the very similar SPACE sequence [53,54].

We cannot explain all the differences in the appearance of the Gradient-Echo and SilentMT images of the boiled egg, in particular the difference in apparent size of the egg; a more thorough investigation including B1 and B0 measurements might shed further light. More structural details were observed in the SilentMT image of the egg compared to the Gradient-Echo equivalent. Cooked egg is often used as a phantom for T2 [46] or MTR measurements [47]. The simplest explanation of this observation is that proteins in the egg decayed during the day it was left at room temperature, and these developed a greatly shortened T2, providing extra contrast which could not be detected by the Gradient-Echo image. The other possibility is that a Magnetisation Transfer effect could be taking place between two very short-T2 signal sources which is not observable with the MT-weighted Gradient Echo [20]. We do not currently have a method of measuring T2 values of less than 10 ms on our scanner, so we cannot verify either of these hypotheses at this time. Further investigations using more complex quantitative Magnetisation Transfer measures might be able to provide full explanations of all the features observed in the SilentMT egg image.

Observations in the MS patient also support our proposal that SilentMT and Silent MTR can detect short-T2 signal that other sequences cannot. We detected one lesion in the MS patient using SilentMT which was not initially observed either on the 7T FLAIR or even on the “reference truth” 1.5T FLAIR. We suspect the lesion had a T2 short enough to make it undetectable by the FLAIR sequences, both of which have long echo times, but we cannot provide a measure of the T2 value of the lesion.

Silent MTR revealed areas of increased MTR in peri-ventricular white matter and in the temporal lobe of the MS patient. No such changes have been observed in healthy controls scanned with this sequence and, in our experience, Silent MTR maps from healthy volunteers are visually similar to MTR maps obtained with conventional sequences at 1.5T and 3T. The adiabatic ASPIR pulse generates uniform MT weighting and MTR maps, giving us confidence that the increased MTR we have observed in the MS patient is not caused by transmit B1 inhomogeneity.

While variations in subject head and neck size can lead to variations in inferior temporal lobe signal, we also saw changes in the superior portion of the temporal lobe, where head/neck position effects should not be a factor.

MTR changes in MS are usually decreases associated with a focal lesion, whereas we observed apparent heterogeneous increases in MTR in a more widespread region. We note this regional variation of Silent MTR changes could be compared to the “MTR gradient” observations made by several other groups using advanced post-processing techniques on conventional MTR maps in large groups of MS patients [14,15,16]. We intend to investigate these unexpected changes more fully in future work.

We have only limited data at this point, so we have by necessity restricted our analysis to qualitative assessments. More subjects and MS patients need to be acquired to build up a fuller picture of the questions raised by these first observations. This study used a “reference truth” measurement using 1.5T FLAIR data taken on a clinical scanner at a time very close to when the 7T data was acquired. Further work would require creation of maps of short T2 to test the hypothesis that SilentMT’s increased sensitivity arises from MR signals with short T2. A study combining such T2 measures with a comparison of MTR maps derived both from Silent and from Gradient-Echo images would provide more information about the origin of the signal changes reported here, and possibly identify possible areas to improve its sensitivity and, perhaps more importantly, its specificity.

It would be interesting to compare SilentMT with other candidate myelination measures such as Quantitative Susceptibility Mapping or Myelin Water Fraction [55]. In order to address transmit B1 inhomogeneity, several groups are now using adiabatic spin-locked pulses to provide magnetisation transfer weighting or related contrasts [40,44] but several important aspects of spin evolution, including direct saturation, have not yet been quantified. Further work should be done to address these questions. Other areas of possible improvement would be the creation of dual-echo SilentMT or related approaches to remove the effect of long-T2 signal contributions as has been done for UTE-based sequences [25,26,27]. We should point out that TE is not well defined either for ZTE in general (it being an FID-based technique) or specifically for the prototype sequence we have used for this work so such modifications would probably not be trivial.

Clinical interest in the important area of in vivo characterisation of short-T2 substances associated with pathology is growing; improvements in MR technology, new acquisition techniques [56,57], and even new MR scanner designs [28] are beginning to address this burgeoning area of research.

## 5. Conclusions

We have investigated the performance of an MT-weighted 3D-radial ZTE sequence (“SilentMT”) at 7T in a biologic egg phantom, a healthy volunteer, and a patient with Multiple Sclerosis. The SilentMT images show good contrast between white matter, grey matter, and CSF, reminiscent of the contrast seen in conventional T2-weighted scans. A qualitative radiologic assessment observed that SilentMT matched the performance of 1.5T FLAIR in detecting MS lesions, and was superior to the 7T FLAIR sequence that we used. One MS lesion was easily detected on the SilentMT, was very hard to see on the 1.5T FLAIR, and was not detected on the 7T FLAIR. Our interpretation is that this MS lesion had a short T2, which FLAIR sequences are less able to detect.

SilentMT images and Silent MTR maps showed robustness against transmit RF inhomogeneities typically observed at 7T.

SilentMT images of a biologic egg phantom showed detailed structures which were not evident on the conventional MT-weighted sequences, suggesting an enhanced sensitivity to pathologic changes associated with short-T2 MR signal sources.

Silent MTR maps of the MS patient showed markedly raised areas of MTR both peri-ventricularly and in regions of the brain close to the cortex, which could be in apparent agreement with “MTR gradient” observations made by other groups with conventional MTR sequences [14].

We believe these preliminary results show the potential of SilentMT, which should be investigated with further studies.

## Data Availability

Raw data for this study cannot be made generally publicly available at this time as it is part of an ongoing larger study.

## Abbreviations

The following abbreviations are used in this manuscript:

MRI	Magnetic Resonance Imaging
MR	Magnetic Resonance
MT	Magnetisation Transfer
ZTE	Zero Time of Echo
UTE	Ultra-short Time of Echo
MS	Multiple Sclerosis
RF	RadioFrequency
TE	Echo Time
MP2RAGE	Magnetisation-Prepared 2 Rapid Gradient-Echo
FLAIR	Fluid Attenuated Inversion Recovery
FSE	Fast Spin Echo
MTR	Magnetisation Transfer Ratio
7T	7 Tesla
SNR	Signal to Noise Ratio
ASPIR	Adiabatic Spectral Inversion Recovery
B1	RF field
B1+	Transmit RF field
B0	Static Magnetic field
TR	Time of Repetition
TI	Inversion Time
